# Resveratrol enhances polyubiquitination-mediated ARV7 degradation in prostate cancer cells

**DOI:** 10.18632/oncotarget.18003

**Published:** 2017-05-19

**Authors:** Sarah Wilson, Lucia Cavero, Dali Tong, Qiuli Liu, Kyla Geary, Nicholas Talamonti, Jing Xu, Junjiang Fu, Jun Jiang, Dianzheng Zhang

**Affiliations:** ^1^ Department of Bio-Medical Sciences, Philadelphia College of Osteopathic Medicine, Philadelphia, Pennsylvania 19131, USA; ^2^ Center for Chronic Disorders of Aging, Philadelphia College of Osteopathic Medicine, Philadelphia, Pennsylvania 19131, USA; ^3^ Department of Urology, Institute of Surgery Research, Daping Hospital, Third Military Medical University, Chongqing 400042, China; ^4^ Key Laboratory of Epigenetics and Oncology, The Research Center for Preclinical Medicine, Southwest Medical University, Luzhou, Sichuan 646000, China

**Keywords:** prostate cancer, ARV7, resveratrol, polyubiquitination, castration resistance

## Abstract

Although androgen deprivation therapy (ADT) serves as the primary treatment option for localized or metastatic prostate cancer, most cases eventually develop into castration-resistant prostate cancer (CRPC). However, androgen receptor (AR) continues to be functional in CRPC through various mechanisms, including the development of AR splicing variants, especially ARV7. Since it lacks the ligand binding domain but retains the intact DNA binding domain, ARV7 is constitutively active, which makes ARV7-positive prostate cancer responsive to neither abiraterone nor enzalutamide. In this study, we explored the effect of resveratrol on ARV7 transcriptional activity and the potential for development of resveratrol as a treatment for ARV7-positive prostate cancer. First, we ectopically expressed ARV7 in PC3 cells, an AR-negative prostate cancer cell line, and demonstrated that resveratrol is capable of inhibiting ARV7 transcriptional activity by downregulating ARV7 protein levels. Of note, resveratrol does not affect the mRNA levels of ARV7 nor its nuclear translocation. Next, we demonstrated that resveratrol is capable of downregulating the levels of the endogenously expressed ARV7 as well as AR target gene mRNAs in 22RV1 prostate cancer cells. Mechanistically, resveratrol downregulates ARV7 by enhancing ARV7 polyubiquitination and subsequent proteasome-mediated degradation. These findings suggest that resveratrol could be a potential treatment for ARV7-positive CPRC.

## INTRODUCTION

Prostate cancer is one of the most frequently diagnosed cancers and the second leading cause of cancer-related death in men in the United States [1, 2]. In addition, the incidence of prostate cancer is increasing significantly due to the effects of the western lifestyle and the increase of the aging population [3, 4]. Androgen-deprivation therapy (ADT) is the first line of defense, and most of the time, an effective treatment for localized and metastasized prostate cancers. However, a quarter of these patients suffer relapse of the disease and most prostate cancers eventually become castration resistant (CRPC) with tumor recurrence and metastasis within a period of 6 months to 3 years [1, 5, 6]. Furthermore, in the less developed countries, most prostate cancers are diagnosed at advanced stages [7]. Therefore, prostate cancer is a big burden for both developed and developing countries, and thus understanding of the mechanisms underlying prostate cancer development and progression as well as developing efficacious therapies for prostate cancer, especially CRPC, is urgently needed.

The androgen/androgen receptor (AR) axis plays crucial roles in both normal prostate function and the development of prostate diseases including prostate cancer. Depleting androgen or inhibiting AR transcriptional activity has been proven to be effective in prostate cancer treatment. Multiple lines of evidence show that re-establishment of functional AR transcriptional activity is a critical event in the emergence of CRPC. Both abiraterone and enzalutamide are currently the best agents for CYP17 and AR inhibition, respectively. These drugs either individually or in combination can extend the survival of patients with CRPC. However, development of resistance to these drugs is inevitable [8, 9] for a subgroup of CRPC expressing different AR variant characterized with the loss of the c-terminal domain but maintaining both N-terminal transcriptional domain and the ligand binding domain [10, 11]. Reports from different groups showed that prostate cancer cells expressing constitutively active AR splicing variants respond to neither of these reagents [8, 12, 13].

More recent data suggest that among the 22 AR slicing variants identified so far [14], ARV7 is not only the most prevalent [15, 16] but also most relevant to the development of CRPC [17]. ARV7 is generated from alternative splicing of the third intron of the AR pre-mRNA, which creates a cryptic exon and leads to the formation of a truncated AR with a stretch of 16 extra amino acids unique for this particular variant. Although ARV7 is expressed in the presence of AR-full length mRNA and most of the time co-expressed with full-length AR, results from different labs showed not only that the positivity but also the intensity of ARV7 are enhanced by hormonal therapies. For example, Antonarakis et al [18] reported that ARV7 was detected in 55% of patients who had received prior abiraterone versus those who had not; enzalutamide treatment increased ARV7 expression from 15% to 50%, suggesting that ADT can enhance the expression of ARV7 to a certain degree. More importantly, clinical responses were inversely correlated with ARV7 expression: 68% of ARV7-negative patients showed PSA response when treated with abiraterone comparing to 0% response in the ARV7-positive group. When treated with enzalutamide, 53% and 0% of patients showed PSA response in the ARV7-negative and ARV7-positive groups, respectively [17]. Therefore, the identification and application of agents capable of targeting ARV7 would be a valuable strategy in treatment of CRPC.

Resveratrol (RSV, 3, 4′,5-trihydroxystilbene) is one of the well-documented reagents in prostate cancer chemoprevention [19]. Chemically, RSV is a polyphenol transhydroxystilbene found at high levels in grapes and red wines [20]. Animal studies have demonstrated that RSV is rapidly absorbed by the gut and distributed into different tissues [21, 22]. Since the first reported cancer chemopreventive effects of RSV in 1997 [23], both epidemiological and case-controlled studies have demonstrated that RSV and/or consumption of high RSV-containing foods and drinks can reduce prostate cancer incidences [24]. There are multiple lines of evidence shown that RSV exerts its effects on prostate cancer in an AR-dependent manner [25–27] through involvement of the regulation of AR expression and function [28, 29]. It has been well established that RSV downregulates the expressions of both AR and AR target genes [30–32]. Harada *et al*. reported that RSV represses AR target gene expression, at least partially, by enhancing AR degradation in a time- and dose-dependent manner [33]. We have demonstrated that RSV regulates AR target gene expression by repressing AR transcriptional activity without affecting AR nuclear translocation nor DNA binding [34].

In this report, we showed that RSV is capable of inhibiting ARV7-mediated transcription of AR target genes in prostate cancer cells by downregulating the protein levels of both ectopically and endogenously expressed ARV7 without affecting either mRNA levels nor its nuclear translocation. Mechanistically, RSV enhances polyubiquitination and subsequent degradation of ARV7 in a proteasome-dependent manner.

## RESULTS

### Ectopically expressed ARV7 is capable of upregulating PSA in PC-3 cells

**Figure 1:**
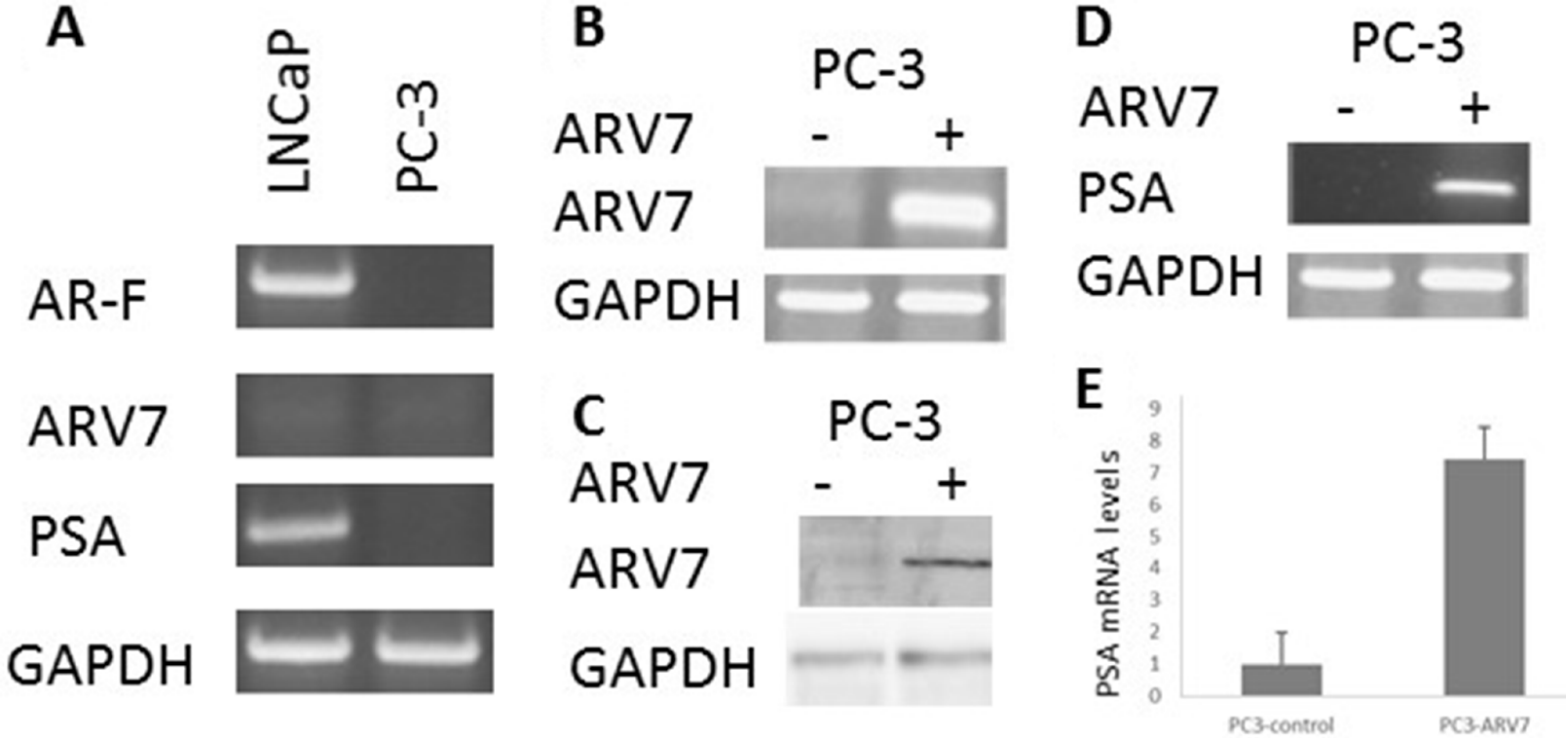
Ectopically expressed ARV7 in PC-3 cells is capable of upregulating PSA (**A**) RT-PCR with mRNAs from both LNCaP and PC-3 cells with primers specific for full-length AR, ARV7, PSA and GAPDH. (**B**–**C**) PC-3 cells were transiently transfected with or without plasmid expressing ARV7, and RT-PCR (B) or WB (C) was conducted. (**D**–**E**) RT-PCR (D) or qPCR (E) was conducted with mRNAs from PC-3 cells transfected with or without ARV7.

Since it has been reported that the transcriptional activity of ARV7 is partially dependent on the expression of full-length AR [35], we wanted to determine if ARV7 is capable of regulating AR target gene expression in a full-length AR-free prostate cancer cellular environment. To do so, we decided to ectopically express ARV7 in PC3 cells, a prostate cancer cell line expressing neither full-length AR nor ARV7, by transiently transfection of plasmids expressing ARV7. To confirm that PC3 cells are ARV7- and PSA-negative, we conducted RT-PCR with mRNAs obtained from two prostate cancer cell lines PC3 and LNCaP. Since LNCaP cells were known AR-positive prostate cancer cells and express readily detectable PSA, we chose to use LNCaP cells as the positive control. As expected, both full-length AR and PSA were readily detectable by RT-PCR but with undetectable level of ARV7 in LNCaP cells. However, none of the full-length AR, PSA or ARV7 was expressed in PC3 cells (Figure 1A). Then, PC3 cells were transiently transfected with or without plasmid expressing ARV7 followed by RT-PCR (Figure 1B) and western blot (Figure 1C) assays. As shown in Figure 1B and 1C, ARV7 was successfully transfected and expressed in PC3 cells, as evidenced by the fact that ARV7 mRNA (Figure 1B) and protein (Figure 1C) were only detected in the cells transfected with ARV7-expressing plasmid. In addition, both RT-PCR (Figure 1D) and quantitative PCR (Figure 1E) demonstrated that the ectopically expressed ARV7 is capable of upregulating AR target gene PSA. These data also suggest that ARV7 can function without the full-length AR.

### RSV inhibits ARV7 transcriptional activity by downregulating its protein levels

**Figure 2:**
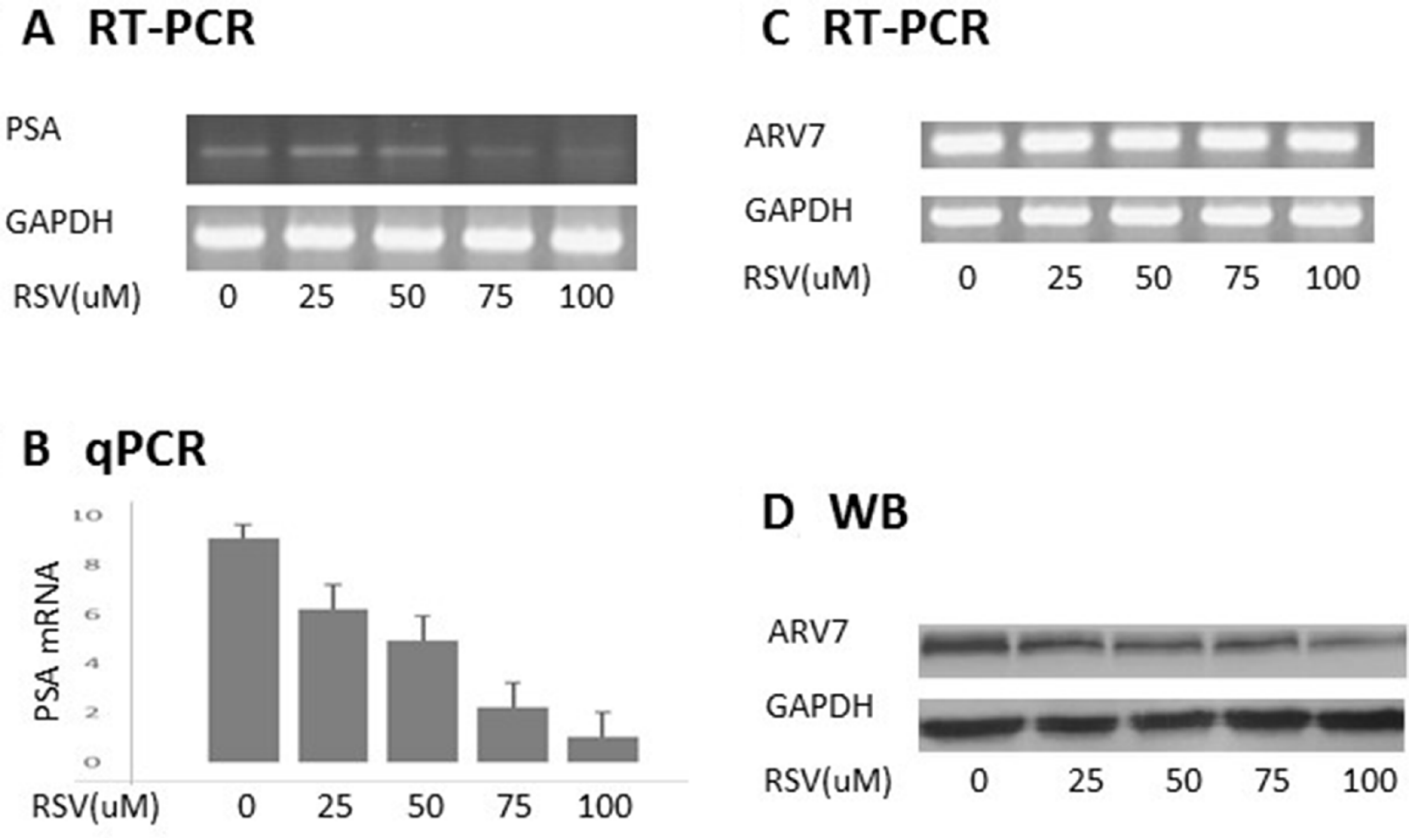
RSV inhibits ARV7 transcriptional activity by downregulating ARV7 protein levels PC-3 cells were transiently transfected with plasmid expressing ARV7 and treated with different concentrations (0–100 μM) of RSV for 24 hours. Both RT-PCR (**A**) and qPCR (**B**) were conducted to estimate the effect of RSV on the mRNA levels of PSA; RT-PCR and WB were conducted to estimate the levels of ARV7 mRNA (**C**) and protein (**D**).

To determine if RSV can inhibit ARV7 transcriptional activity, we treated PC3 cells transfected with ARV7-expressing plasmid with different concentrations of RSV for 24 hours. Total mRNAs were purified and the PSA levels serving as an indicator of the ARV7 transcriptional activity were estimated by RT-PCR (Figure 2A) and qPCR (Figure 2B). Figure 2A and 2B show that ARV7 transcriptional activity is inhibited by RSV in a dose-dependent manner. Next, we conducted RT-PCR and western blot assays to determine if RSV downregulates ARV7 transcriptional activity by repressing its mRNA or protein levels. As shown in Figure 2C, ARV7 mRNA levels were not affected by RSV treatment but protein levels were downregulated by RSV in a dose-dependent manner (Figure 2D). These data collectively demonstrated that RSV is capable of inhibiting ARV7 transcriptional activity in a dose-dependent manner and mechanistically downregulating ARV7 protein levels without affecting mRNA level.

### RSV does not affect ARV7 nuclear translocation

**Figure 3:**
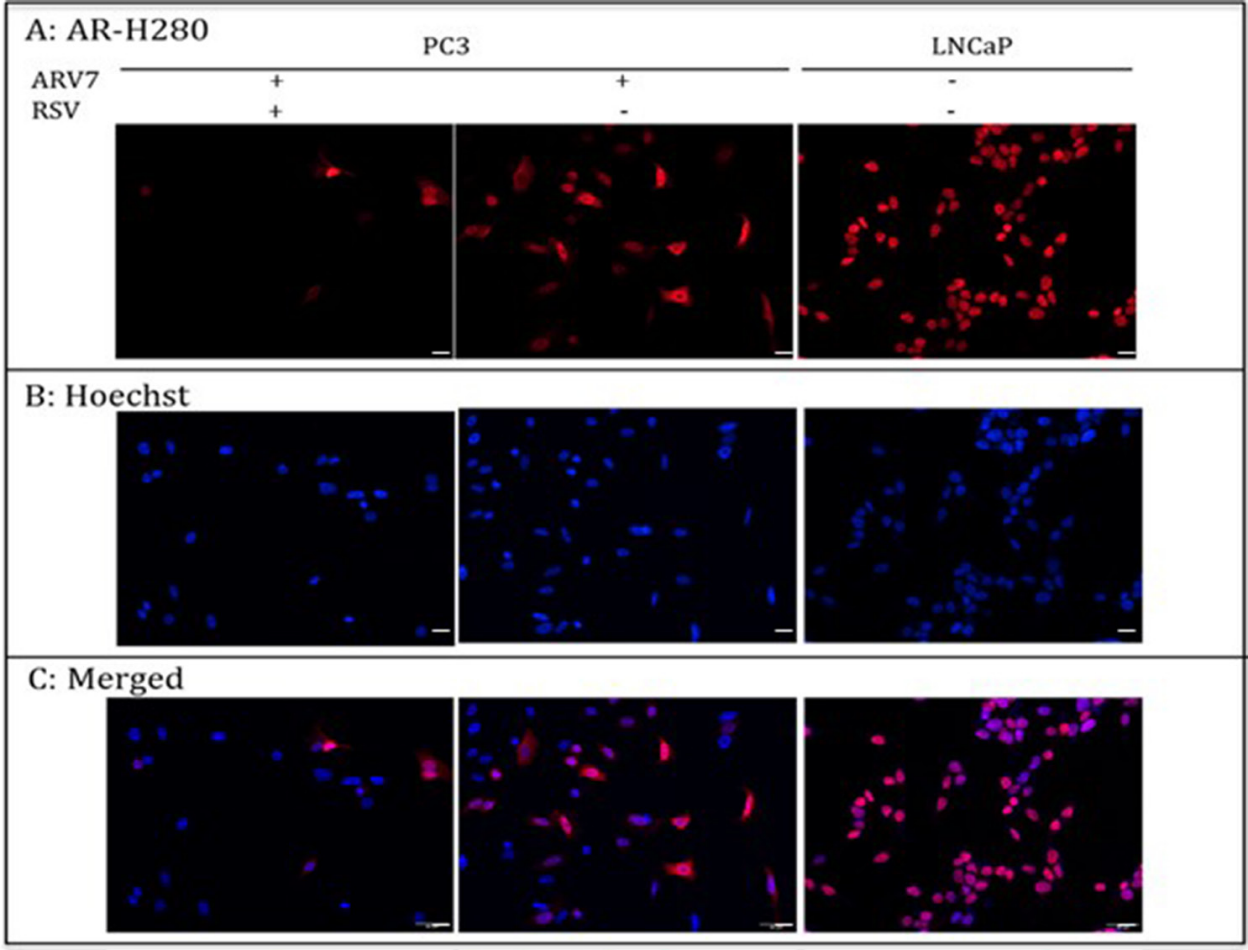
PC-3 cells were transiently transfected with plasmid expressing ARV7 and treated with or without RSV for 24 hours LNCaP cells were treated with 10 nM R1881 for 24 hours. Immunostaining was conducted to determine the subcellular localizations of AR and ARV7. Scale bar: 100 μm.

Upon the binding of androgens, the full-length AR translocates to the nucleus from the cytoplasm and interacts with the androgen responsive element (ARE) to regulate AR target gene expression. Therefore, targeting AR's nuclear translocation can be used as a strategy in repressing AR transcriptional regulation. We wanted to determine if RSV represses ARV7 transcriptional activity by inhibiting its nuclear translocation. To this end, we treated the PC3 cells transfected with ARV7-expressing plasmid with or without 100 μM RSV for 24 hours and conducted immunostaining with antibody against the N-terminus of AR, which is able to recognize both full-length AR and ARV7. Immunostaining of LNCaP cells treated with the synthesized androgen R1881 serves as experimental control. As shown in Figure 3, although RSV treatment reduced the total ARV7 levels dramatically (consistent with changes observed in Figure 2), the expressed ARV7 was mainly found in the nuclei whether the cells were treated with or without RSV. These data suggest that RSV inhibits ARV7 transcriptional activity by downregulating ARV7 protein levels without affecting its nuclear translocation. In addition, since ARV7 was ectopically expressed in PC3 cells without the full-length AR, we also conclude that the nuclear translocation of ARV7, at least for the ectopically expressed form, is independent of the full-length AR.

### RSV downregulates endogenously expressed ARV7 without affecting full-length AR

**Figure 4:**
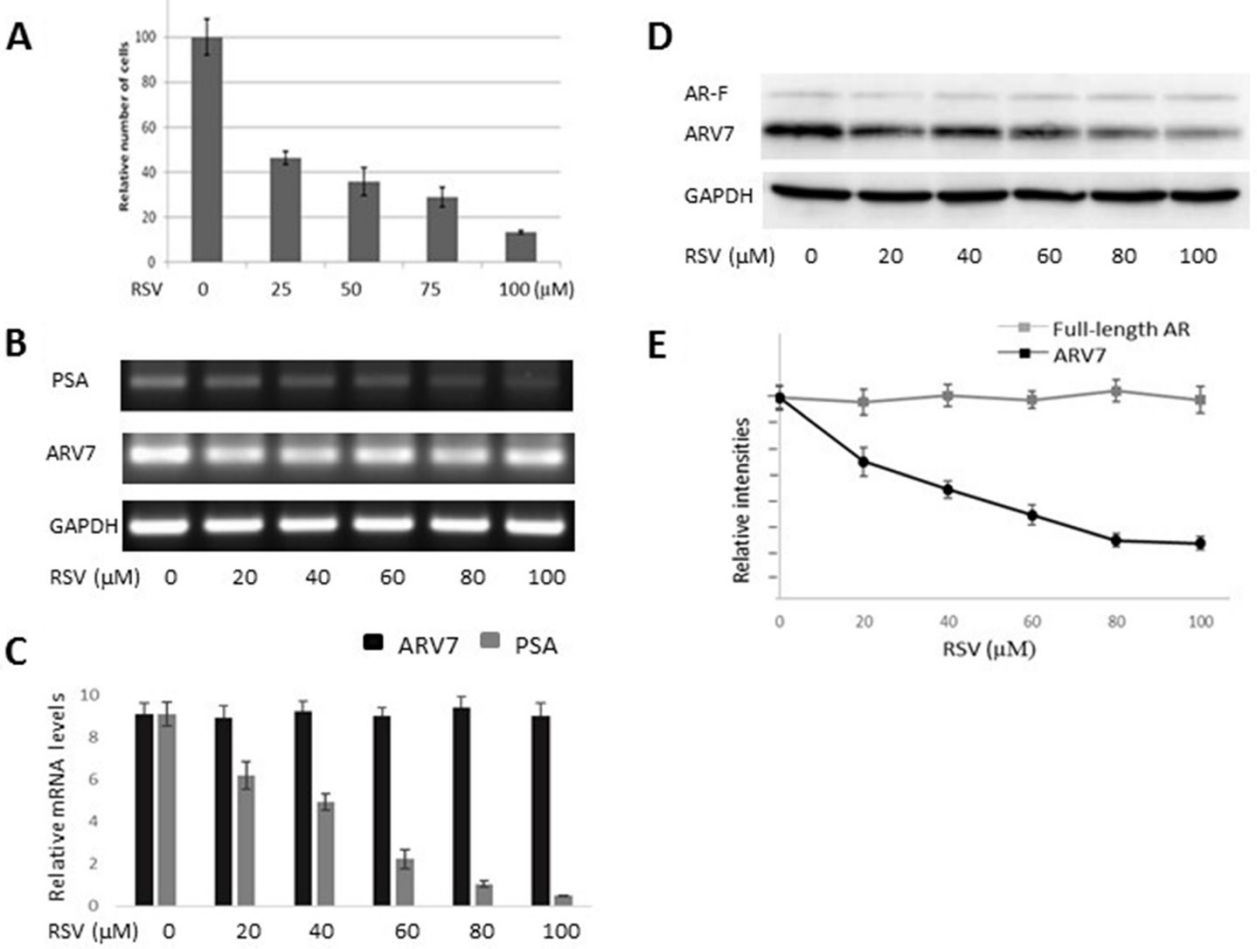
RSV inhibits prostate cancer growth and downregulates ARV7 in 22RV1 cells without affecting full-length AR 22RV1 cells were treated with different concentrations (0–100 μM of RSV for 24 hours and the number of viable cells were estimated through cell counting (**A**). The mRNA levels of PSA and ARV7 were estimated by RT-PCR (**B**) and q-PCR (**C**). The protein levels of ARV7 and full-length AR were estimated by western blotting assays (**D** and **E**).

After demonstrating RSV's effects on the ectopically expressed ARV7, we wanted to determine if RSV has similar effects on ARV7 endogenously expressed in prostate cancer cells. We chose to use the 22RV1 cell line because it represents the CRPC prostate cancer cells and both full-length AR and ARV7 were expressed endogenously in these cells. First, we treated 22RV1 cells with increasing concentrations of RSV for 24 hours, and the cell viabilities were estimated by counting the number of live cells. As shown in Figure 4A, RSV is capable of inhibiting 22RV1 cell growth in a dose-dependent manner. Then, we conducted RT-PCR to estimate RSV's effects on the mRNA levels of PSA and ARV7. Figure 4B (RT-PCR) and 4C (qPCR) show that RSV is able to dose-dependently repress PSA levels without affecting the levels of ARV7 mRNA. In addition, we have conducted RT-PCR on another three potential ARV7 target genes (UBE2C, TMPRSS2 and FKBP51) to estimate RSV's effect on ARV7 transcriptional activity. Supplementary Figure 1 show that the mRNA levels of all of them were downregulated by RSV. However, western blotting assays demonstrated that the ARV7 protein levels were dose-dependently downregulated by RSV (Figure 4D and 4E). Of note, the protein levels of the full-length AR were not affected by RSV treatment in these cells. These data suggest that resveratrol is capable of downregulating ARV7 protein levels without affecting the full-length AR protein level in 22RV1 cells.

### RSV enhances ARV7 protein degradation in prostate cancer cells

**Figure 5:**
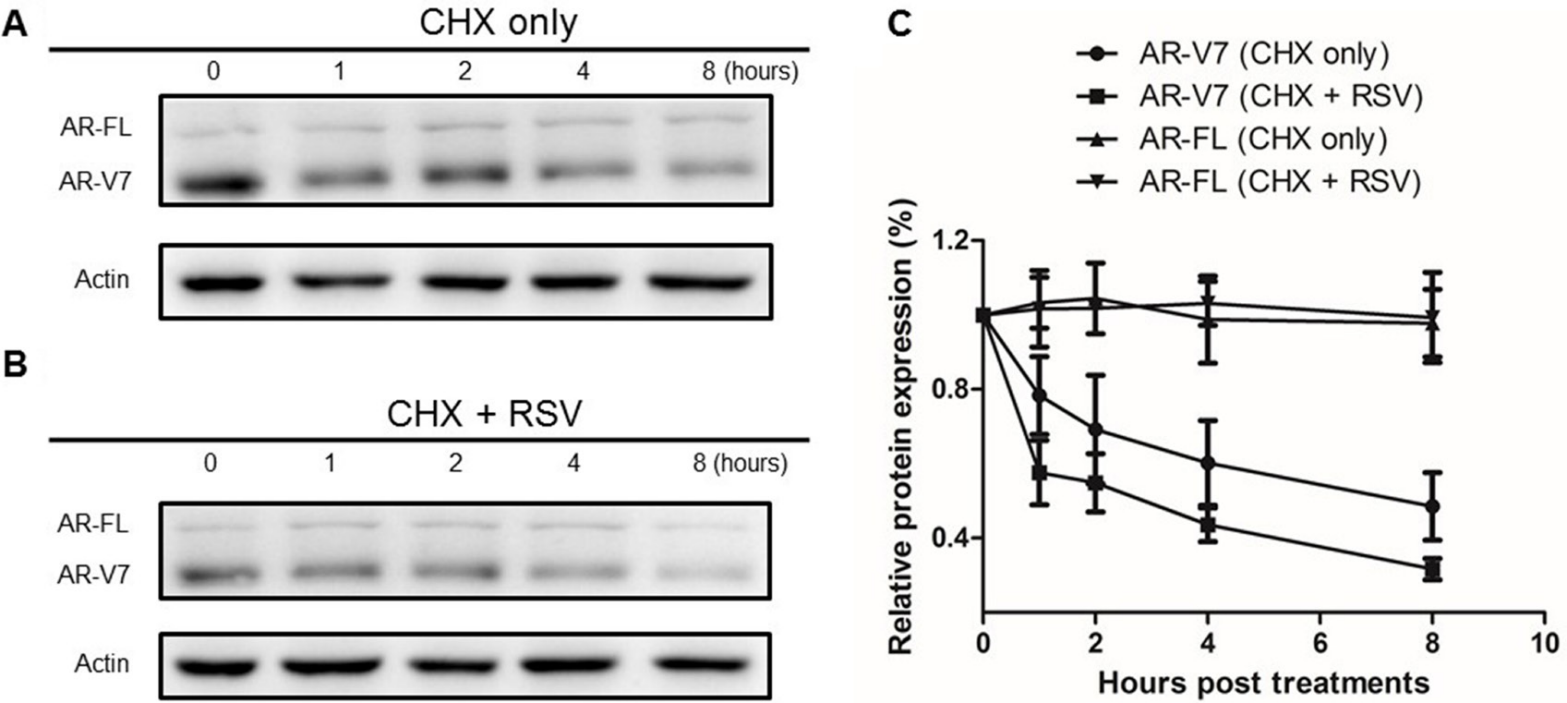
Effect of RSV on ARV7 protein degradation 22RV1 cells were cultured in growth media with 50 nM CHX along (**A**) or in combination with 100 μM RSV (**B**) and collected at different time points (0–8 h). Western blotting assays were conducted with antibodies against ARV7 and actin. The intensities of the protein bands were estimated by Image-J and normalized with that of actin and plotted (**C**).

Next, we wanted to understand the mechanism in RSV-induced ARV7 protein downregulation. To this end, we treated 22RV1 cells with 50μM/mL of cyclohexmide alone or in combination with 100μM RSV for different time periods. Cells were collected at different time points and both full-length AR and ARV7 protein levels were determined by western blotting assays with antibody against either AR or actin. As shown in Figure 5A and 5B, ARV7 protein levels decreased in a time-dependent manner in either treatment but the full-length AR and actin levels were relatively unchanged during the observed period. However, compared to the cells treated with cyclohexmide alone, RSV appears to be able to reduce ARV7 stability because ARV7 levels decreased dramatically in the cells treated with the combination of RSV and cyclohexamide. The intensities of the protein bands were estimated by ImageJ program and the estimated full-length AR and ARV7 levels were normalized with actin, and the normalized levels in both treatments were plotted (Figure 5C). It is clear that RSV is capable of enhancing ARV7 degradation by reducing the half-life of ARV7 from about 4 hours (CHX only) to 2 hours (CHX+RSV). Interestingly, the protein levels of full-length AR were not affected significantly.

### RSV enhances ARV7 polyubiquitination and subsequent degradation

**Figure 6:**
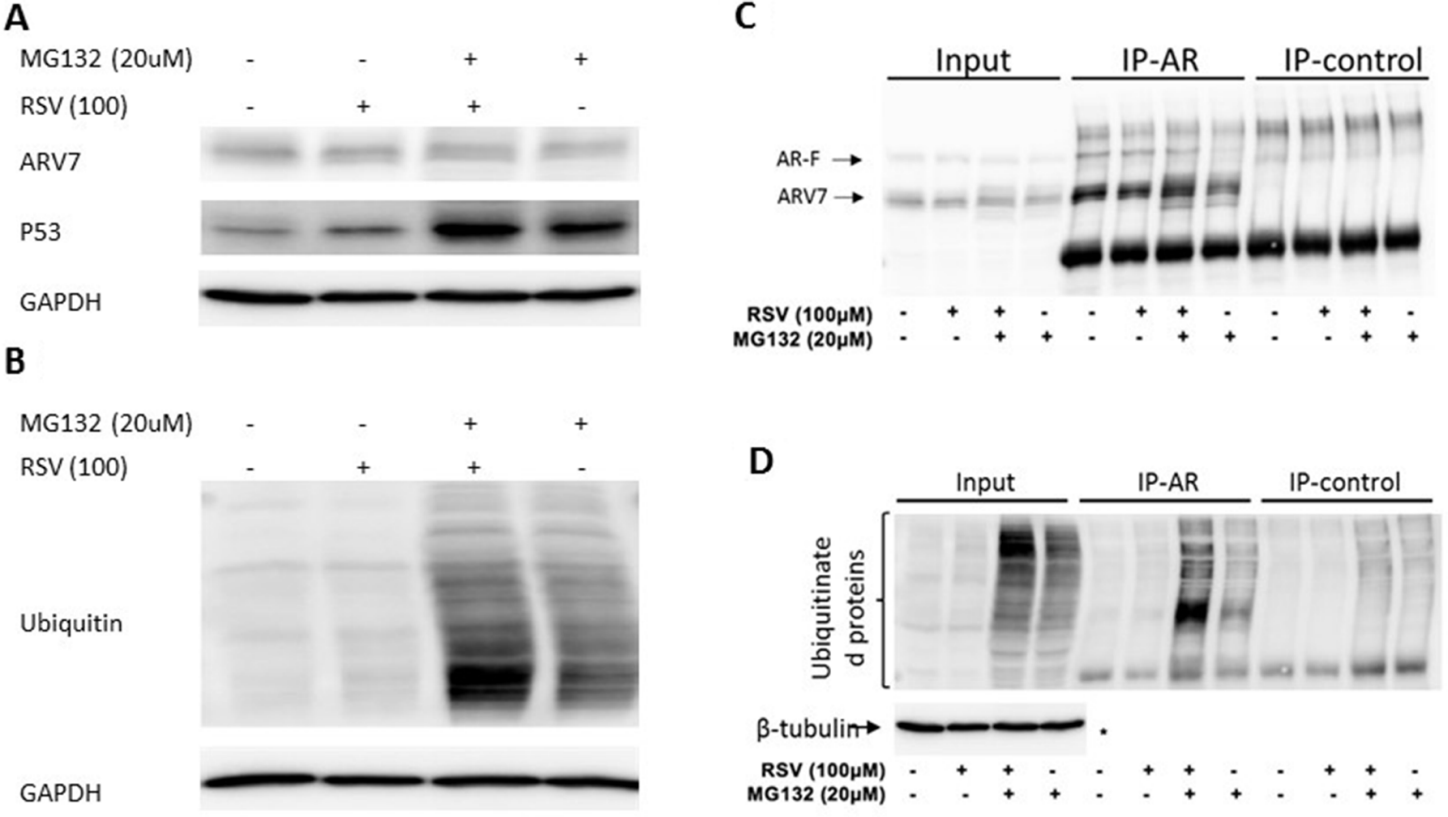
Effect of RSV on polyubiquitination in prostate cancer cells (**A**–**B**) 22RV1 cells were treated with or without RSV and MG132 for 24 hours. WB was conducted to estimate the levels of ARV7, P53 (A) and ubiquitinated proteins (B). Lysates were used for immunoprecipitation with antibodies against AR or nonspecific IgG, and the precipitated proteins were analyzed by WB with antibodies against AR (**C**), ubiquitin or tubulin (**D**).

To test if RSV enhances ARV7 degradation through the polyubiquitination-mediated proteasome pathway, we treated the 22RV1 cells with RSV or MG132 alone or in combination for 24 hours. ARV7 protein levels were estimated by western blot assays with p53 as the experimental control. As shown in Figure 6A the p53 level was elevated when the cells were treated with either RSV or MG132 alone or in combination. However, the ARV7 levels were not significantly affected under the same conditions. In addition, treatment of the cells with RSV alone reduced the ARV7 level as seen previously. These data suggest that RSV's effect on ARV7 degradation is either not through the polyubiquitination-mediated pathway or somehow different from the mechanism used by p53 degradation. To determine if RSV affects polyubiquitination in general, we conducted western blot assay using antibody against ubiquitin. As shown in Figure 6B, although the overall levels of polyubiquinated proteins in these cells were not affected by RSV treatment alone (lane 2), RSV is capable of enhancing the MG132 inhibited protein degradation (lane 3). As expected, when the cells were treated with MG132 alone the polyubiquitinated protein levels were elevated (lane 4). These data suggest that RSV is capable of enhancing protein ubiquitination but the polyubiquitinated proteins were quickly degraded in the absence of MG132.

We then treated 22RV1 cells with RSV or MG132 alone or in combination for 24 hours and conducted immunoprecipitation with antibody against AR N-terminus which is able to recognize both full-length AR and ARV7. Normal IgG was used as a negative control. The precipitated proteins were analyzed by western blotting assays. As shown in Figure 6C, ARV7 was readily detectable in the inputs, and both the full-length AR and ARV7 were successfully precipitated by the specific antibody; the normal IgG only nonspecifically precipitated some background levels of proteins. Of note, either RSV or MG132 alone or in combination failed to affect ARV7 levels significantly. However, when the same blot was stripped and re-probed with antibody against ubiquitin (Figure 6D), we found that MG132 alone was able to increase the levels of polyubiquitinated ARV7 and this effect was synergized with RSV. Nevertheless, the levels of polyubiquitinated ARV7 in the cells treated with RSV alone were about the same as those of the control. These data altogether demonstrate that RSV is capable of enhancing ARV7 polyubiquitination but the polyubiqitinated ARV7 is quickly degraded (RSV alone) by the proteasome system because inhibition of proteasome-mediated protein degradation by MG132 leads to the accumulation of polyubiquitinated ARV7 (RSV+MG132).

Given the fact that 22RV1 cells express both full-length AR and ARV7 and there are potential interactions between them.

In order to demonstrate that RSV mediates ARV7 degradation by enhancing polyubiquitination, similar experiments were done with the exogenously expressed ARV7 in PC3 cells. First, we conducted immunoprecipitation of the exogenously expressed ARV7 in PC3 cells treated with RSV and MG132 alone or in combination (Supplementary Figure 2) and found that RSV downregulated ARV7, MG132 failed to counteract RSV effect. Supplementary Figure 3 shows that, similar to was on endogenously expressed ARV7, the polyubiquitination of the exogenously expressed ARV7 is enhanced by RSV. To further that RSV effect on ARV7 degradation is through polyubiquitination pathway, we estimated the levels of ARV7 and overall protein ubiquitination in 22RV1 cells treated with either RSV or PYR-41 (an inhibitor of the E1 enzyme in the ubiquitination pathway) alone or in combination. Supplementary Figure 4A shows that the total protein levels were comparable among the differently treated samples. However, WB results (Supplementary Figure 4B) indicate that RSV is capable of enhancing polyubiquitinated (comparing lanes 1 and 2) and the E1 enzyme inhibitor PYR-41 effectively repressed RSV-induced poly-ubiquitination (comparing lanes 2 and 3).

## DISCUSSION

**Figure 7:**
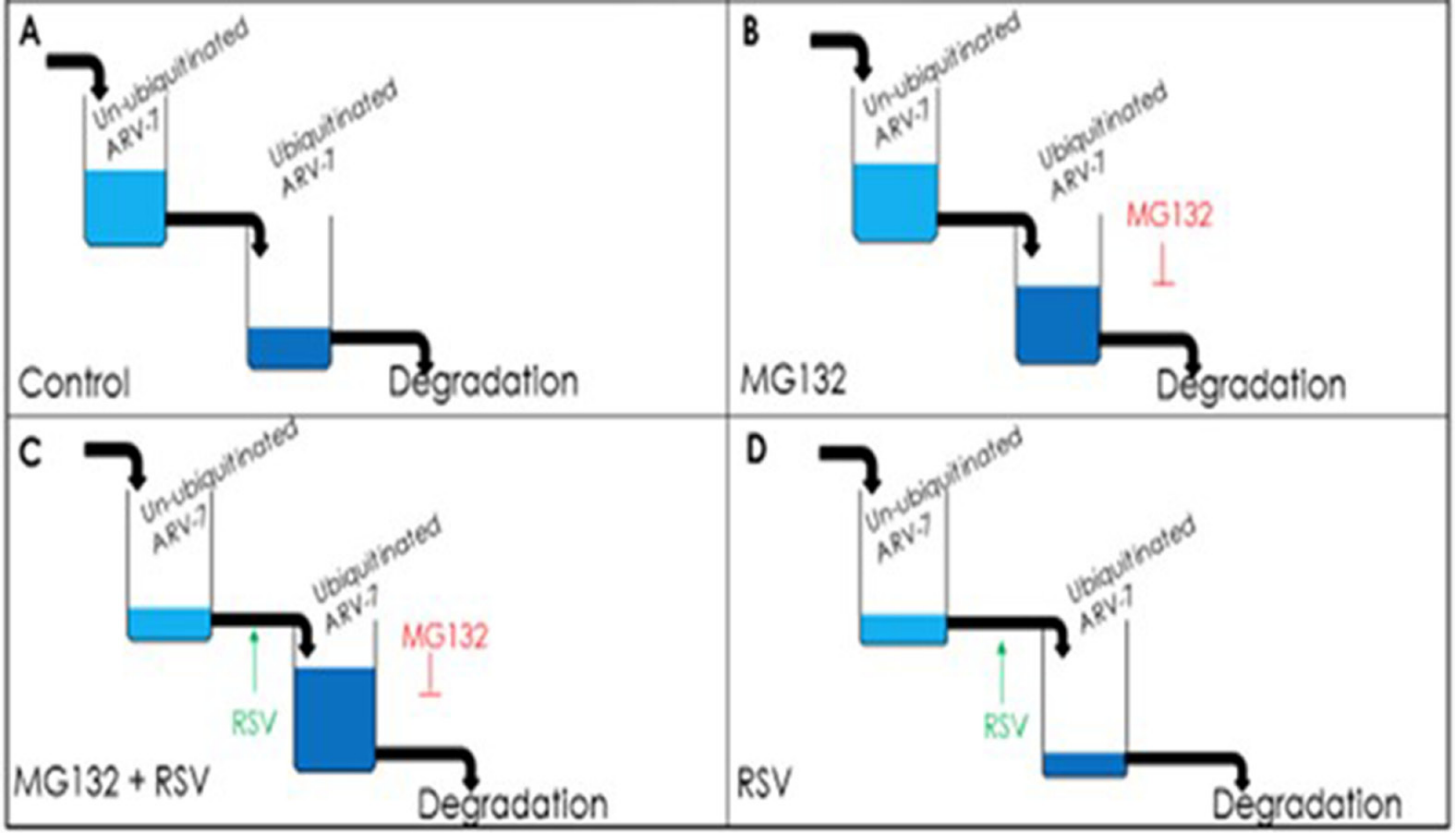
Model of RSV enhancing polyubiquitination-mediated ARV7 degradation

Given the fact that [1] ARV7 positivity strongly correlates with both CRPC status and poor prognoses for prostate cancer patients diagnosed at late stages [36], and [2] ARV7-positive prostate cancers have proven to be resistant to almost all currently available prostate cancer drugs and therapies [37–39], either reducing the level or inhibiting the activity of ARV7 would be plausible therapeutic strategies for ARV7-positive CRPC. Based on this, we evaluated if RSV is able to affect ARV7 and found that RSV is not only capable of downregulating the levels of ARV7 but also of repressing the expression of its target genes in prostate cancer cells. Based on our observations, we proposed a working model (Figure 7) to explain the RSV-enhanced polyubiquitination and subsequent degradation of ARV7 in prostate cancer cells. Without the presence of protein degradation inhibitor MG132, RSV-enhanced ARV7 polyubiqitination is followed by quick degradation. In the presence of MG132, ARV7 is polyubiquitinated but fails to be degraded and therefore the level of polyubiquitinated ARV7 is increased. However, under either condition, the non-ubiquitinated ARV7 is reduced by RSV.

In order to determine if RSV can target ARV7 specifically, we first tested the effects of RSV on the ectopically expressed ARV7 in PC3 cells. Since PC3 cells express neither full-length AR nor AR target PSA (Figure 1), this enabled us to determine the effects of RSV on the ectopically expressed ARV7 in a prostate cancer cellular environment but without the effects of the endogenously expressed ARs. We demonstrated that RSV is capable of downregulating both ARV7 protein levels and its transcriptional activity without affecting its mRNA levels (Figure 2). Note, the transcription of ARV7 in this system was controlled by the CMV promoter and since the level of ARV7 mRNA is not affected by RSV, it suggests that RSV-mediated downregulation of ARV7 protein level is likely not through an effect on ARV7 mRNA stability. The same findings were confirmed with the endogenously expressed ARV7 in 22RV1 prostate cancer cells (Figure 4). Although there are some reports suggesting that a functional ARV7 depends on the existence of the full-length AR [35], our results suggest that ARV7 can regulate AR target gene PSA in a full-length AR-independent manner. In addition, even though it does not contain the canonical nuclear translocation signal, ARV7 is mainly expressed in the nuclei of the prostate cancer cells, and RSV appears not to affect ARV7 nuclear translocation (Figure 3).

It has been well documented that RSV can inhibit the function of the full-length AR through different mechanisms, including enhancement of AR degradation. However, we found that RSV is capable of downregulating ARV7 protein levels without affecting the full-length AR in 22RV1 cells. This observation leads us to speculate that RSV exerts its degradation effects on the full-length AR and ARV7 through different mechanisms. This speculation has been further substantiated by the fact that the half-life of ARV7 but not that of the full-length AR was shortened by RSV in 22RV1 cells. Given the fact that ARV7 has a sequence identical to the full length AR except for the extra 16 amino acids at its C-terminus, we speculate that this short sequence in itself or together with part of the rest of ARV7 plays an essential role in RSV-mediated ARV7 degradation. In addition, this short sequence may also be responsible for the nuclear translocation of ARV7. However, bioinformatics analyses of this short sequence (unpublished data) failed to identify any motif particularly related to protein degradation or nuclear translocation.

It has been reported recently that by screening a drug library containing about 1120 FDA-approved chemicals with an unbiased strategy, the well-studied anti-helminthic drug niclosamide was found to be able to repress ARV7 recruitment to the PSA promoter, resulting in decreased PSA transcription. Further exploration found that niclosamide utilizes a proteasome-dependent pathway to degrade ARV7 protein which resulted in decreased ARV7 protein level but without affecting its mRNA level [40]. Therefore, the authors suggest that niclosamide alone or in combination with other anti-cancer drugs could be used for the treatment of CRPC. However, niclosamide has been used as an anti-helmithic drug with multiple issues unrelated to prostate cancer. Therefore, treating prostate cancers with niclosamide may lead to some undesirable side effects, and thus making this drug a relatively unattractive therapeutic option for CRPC. Although RSV downregulates ARV7 in a similar manner as niclosamide, RSV has not shown any side-effects but with multiple beneficial effects on skin disorders, type II diabetes, cardiovascular diseases, and obesity [41]. Therefore, RSV appears to be superior to niclosamide in downregulating ARV7. However, the drawback to RSV as a potential therapeutic option is its relatively low bioavailability in human tissues [42]. On the other hand, pterostilbene (PTER) is a structural analog of RSV with greater tissue bioavailability than RSV [43]. Our preliminary data suggest that PTER functions similarly to RSV in regard to ARV7 (unpublished data) and once this is fully confirmed, PTER could be an even better drug to target ARV7 in CRPC. In addition, there are other strategies targeting the levels of ARV7. For example, Jin et al. found that NF-kB can increase ARV7 expression in prostate cancer cells. They also found that when NF-kB signaling pathway is blocked, both ARV7 mRNA and protein levels were down-regulated. Furthermore, blocking NF-kB signaling makes CRPC cells become ADT-responsive [44]. Therefore, a combination of therapies targeting ARV7 mRNA levels (such as blocking NF-kB) with RSV and/or PTER could be more efficacious in the treatment of ARV7-positive CRPC.

In summary, we demonstrated that RSV is capable of downregulating both ectopically and endogenously expressed ARV7 in prostate cancer cells without affecting the mRNA levels or the nuclear translocation of ARV7. Mechanistically, RSV enhances ARV7 polyubiquitination and subsequent proteasome-mediated degradation. These observations suggest that RSV possesses a great potential to become a treatment for ARV7-positive CPRC.

## MATERIALS AND METHODS

### Cell lines and chemicals

DMSO, resveratrol (RSV), MG132, R1881 were all purchased from Sigma-Aldrich (St. Louis, MO). Resveratrol stock solution (1 mM) was made by dissolving RSV in DMSO and stored at −20°C in the dark. Antibodies against both full-length AR and ARV7 (N-20), ß-tubulin, GAPDH ubiquitin and normal rabbit IgG were purchased from Santa Cruz Biotechnologies and all stored at 4°C. The ARV7 expressing plasmid was a kind gift from Dr. Lu's lab (The James Buchanan Brady Urological Institute and Department of Urology, The Johns Hopkins School of, Baltimore, MD 21287, USA). LNCaP, 22RV1 and PC3 cells were obtained from American Type Culture Collection (ATCC). PC3 cells were cultured in Ham's F-12 with L-glutamine, 10% premium FBS, and 1% Antibiotic-Antimycotic. LNCap and 22RV1 cells were maintained in RPMI 1640 medium supplemented with 10% (wt/vol) fetal bovine serum (FBS) and 1% antibiotics at 37°C under 5% CO_2_.

### Immunostaining

Cells were grown on glass cover slides, fixed with 3.5% formaldehyde for 15 minutes, and permeabilized with 0.02% NP-40 for 1 minute. After blocking with 5% goat serum for 1 hour, cells were incubated with anti-AR antibodies (N20) for 2 hours. The slides were then incubated in donkey anti-rabbit immunoglobin G conjugated with Alexa Fluor 594 for 2 hours. One drop of mounting medium (Fisher Scientific) was added onto each slide and the images were visualized by conventional microscopy.

### Transient transfection

ARV7 expressing plasmid was transiently transfected into PC3 cells using Lipofectamine 2000 reagent according to the manufacturer's instructions (Invitrogen). Transfected cells were allowed to recover for 6 hours before replacing with regular growth media followed by specific treatments for designated time periods. Cells were harvested and used for specific assays. All experiments were conducted in triplicate.

### RNA isolation, RT-PCR and qPCR

Total RNA was isolated using the RNeasy mini kit (Qiagen) according to the manufacturer's specifications. Total RNA from each sample was reverse-transcribed with random primers using a StrataScript reverse transcriptase kit (Stratagene) followed by either semi-quantitative or real-time PCR. Our standard PCR procedures are as follows: in a 25 μl reaction, DNA was denatured at 94°C for 2 min and followed by 30 cycles of 94°C for 45sec; 62°C for 45sec and 72°C for 45sec. After the last cycle, reactions were incubated for an additional 5 min at 72°C to ensure that all DNA strands were extended to the ends. PCR products were separated by electrophoresis on 1% agarose gel and visualized under UV light. The intensities of DNA bands were estimated by the Image-J program. For qPCRs, the SYBR Premix Ex Taq II (Tli RNase H Plus) Kit was used per manufacturer's recommendation.

### Preparation of lysates and western blot

Whole cell lysates were obtained using the nuclear extract kit (Active Motif, California) according the manufacturer's instructions. Protein concentrations were estimated by Bradford reagents and equal amounts of total proteins were separated on a 10% SDS-PAGE gel. The proteins were transferred to nitrocellulose membrane (BioRad, Hercules, CA) using the BioRad Blotting System according to the manufacturer's instructions. Staining with Ponceau Red was done to confirm equal transfer of protein in all lanes. Blots were blocked for 2 hours in 5% non-fat milk and incubated with antiserum overnight at 4°C. After washing three times in TBST, the blots were incubated with the second conjugated antibody. The blots were detected by Supersignal West Pico Chemiluminescent Kit (Pierce). The membranes were then stripped and re-probed for either β-actin or GAPDH as internal controls.

### Immunoprecipitation

Immunoprecipitation was done with cell lysates containing about 400 μg of protein in 100 μL. The lysates were pre-cleared with 25 μL of Pierce Protein A/G Magnetic Beads for 2 h. Lysate + beads were placed on the magnet for 2 min. The pre-cleared lysate was removed from the beads and 1μg of antibody was added. All samples (lysate + antibody) were incubated at 4°C overnight. The following day 100 μL of Pierce Protein A/G Magnetic Beads were added to the lysate + antibody, vortexed and rotated at 4°C for 2 h. All samples were placed on the magnet for 2 min and unbound lysate was removed. Beads were washed 3× with 100 μL washing buffer, vortexed and spun briefly before being placed on the magnet for 2 min. Proteins bound on the beads were eluted with 20 μL of 2× SDS sample buffer, and then boiled for 5 min before electrophoresis.

## SUPPLEMENTARY MATERIALS FIGURES


