# Supplementary material for: Resveratrol enhances polyubiquitination-mediated ARV7 degradation in prostate cancer cells
Source: Oncotarget. 2017 May 19;8(33):54683–93. doi: 10.18632/oncotarget.18003 (PMC5589613; doi:10.18632/oncotarget.18003)
Supplement: Supplementary file 1 [file oncotarget-08-54683-s001.pdf]

Resveratrol enhances polyubiquitination-mediated ARV7 degradation in prostate cancer cells

SUPPLEMENTARY MATERIALS

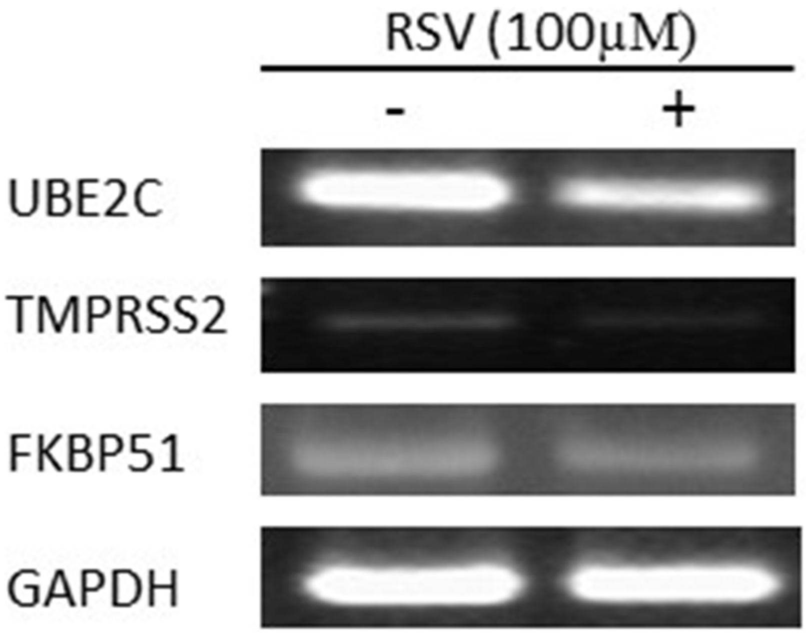

Supplementary Figure 1: The effects of RSV on the mRNA levels of AR target genes. 22RV1 cells were treated with or without 100 µM of RSV for 24 hours. Total RNA was purified, and RT-PCR was conducted with specific primers. The amplified PCR products were separated on 1% agarose gel and visualized under UV light.

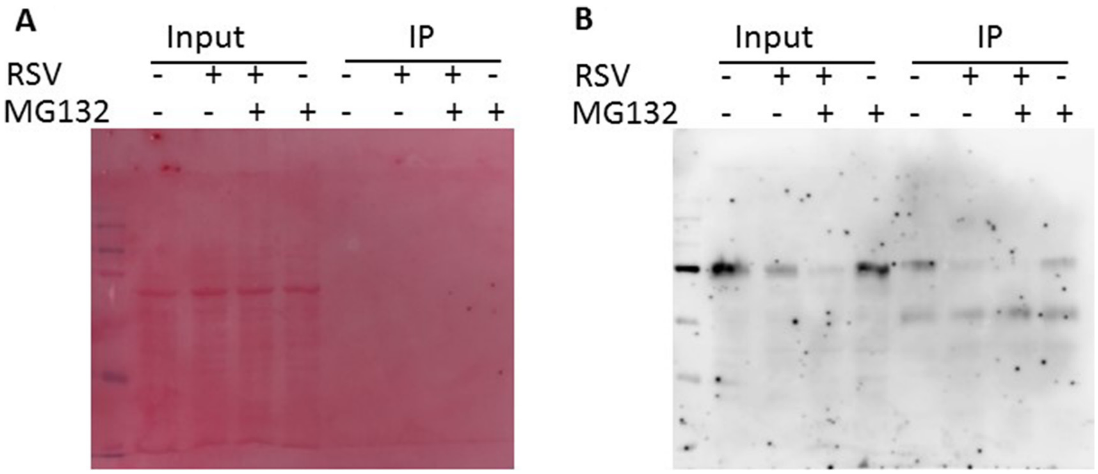

Supplementary Figure 2: Effect of resveratrol on poly-ubiquitination and proteasome-mediated degradation of exogenously expressed ARV7. PC-3 cells were transfected with ARV7-expressing plasmid and cultured for 24 hours. Cells were treated with either resveratrol or MG132 alone or in combination for 24 hours. Cells were lysed in EBC buffer, and cell lysates were used for immunoprecipitation with antibody against the N-terminus of AR. The inputs and IP products were separated on 10% SDS-PAGE and transferred to NC membrane. The Ponceau Red staining (**A**) was followed by WB with antibody against either AR or ubiquitin (**B**).

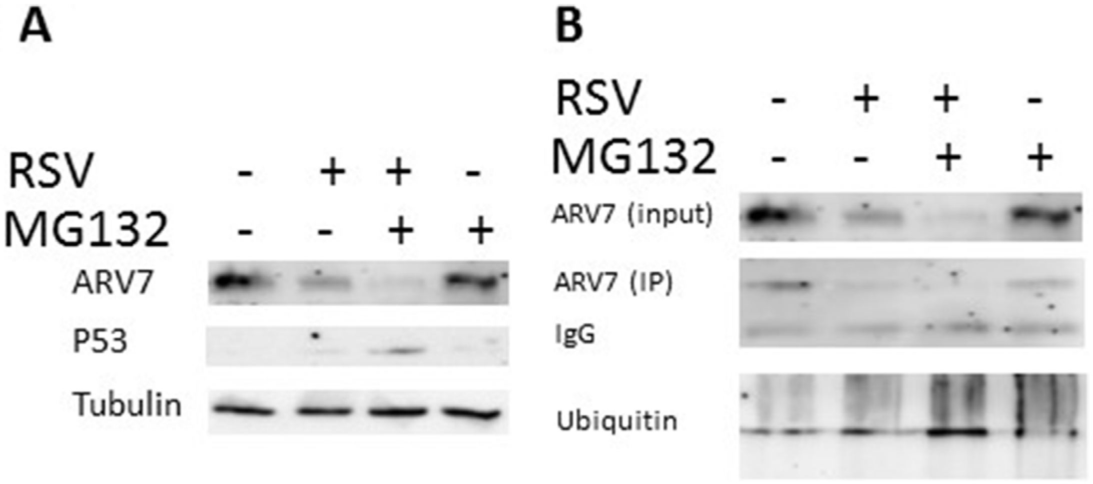

Supplementary Figure 3: Effect of RSV on P53 and ARV7 ubiquitination. (**A**) WB of cell lysates with antibodies against ARV7, p53 and tubulin. (**B**) WB of the immuno-precipitated products with antibodies against ARV7 or ubiquitin.

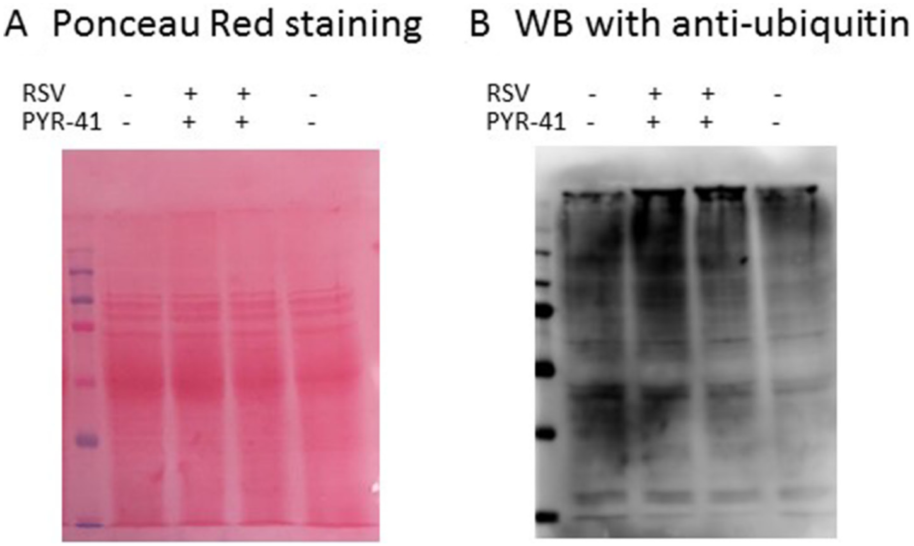

Supplementary Figure 4: The effect of RSV on protein poly-ubiquitination. The 22RV1 cells were treated with either RSV (100 µM) or PYR-41 (10 µM) alone or in combination for 24 hours. Cells were collected and lysed in RIPA buffer. Lysate proteins (100 µg) were separated on SDS-PAGE gel, transferred to NC-membrane and either stained with Ponceau Red (**A**) or analyzed via WB with antibody against ubiquitin.
